# A framework for assessing reliability of observer annotations of aerial wildlife imagery, with insights for deep learning applications

**DOI:** 10.1371/journal.pone.0316832

**Published:** 2025-01-15

**Authors:** Rowan L. Converse, Christopher D. Lippitt, Steven E. Sesnie, Grant M. Harris, Matthew J. Butler, David R. Stewart

**Affiliations:** 1 Center for the Advancement of Spatial Informatics Research and Education, University of New Mexico, Albuquerque, New Mexico, United States of America; 2 Department of Geography and Environmental Studies, University of New Mexico, Albuquerque, New Mexico, United States of America; 3 Division of Biological Sciences, US Fish and Wildlife Southwest Regional Office, Albuquerque, New Mexico, United States of America; Central University of Punjab, INDIA

## Abstract

There is growing interest in using deep learning models to automate wildlife detection in aerial imaging surveys to increase efficiency, but human-generated annotations remain necessary for model training. However, even skilled observers may diverge in interpreting aerial imagery of complex environments, which may result in downstream instability of models. In this study, we present a framework for assessing annotation reliability by calculating agreement metrics for individual observers against an aggregated set of annotations generated by clustering multiple observers’ observations and selecting the mode classification. We also examined how image attributes like spatial resolution and texture influence observer agreement. To demonstrate the framework, we analyzed expert and volunteer annotations of twelve drone images of migratory waterfowl in New Mexico. Neither group reliably identified duck species: experts showed low agreement (43–56%) for several common species, and volunteers opted out of the task. When simplified into broad morphological categories, there was high agreement for cranes (99% among experts, 95% among volunteers) and ducks (93% among experts, 92% among volunteers), though agreement among volunteers was notably lower for classifying geese (75%) than among experts (94%). The aggregated annotation sets from the two groups were similar: the volunteer count of birds across all images was 91% of the expert count, with no statistically significant difference per image (t = 1.27, df = 338, p = 0.20). Bird locations matched 81% between groups and classifications matched 99.4%. Tiling images to reduce search area and maintaining a constant scale to keep size differences between classes consistent may increase observer agreement. Although our sample was limited, these findings indicate potential taxonomic limitations to aerial wildlife surveys and show that, in aggregate, volunteers can produce data comparable to experts’. This framework may assist other wildlife practitioners in evaluating the reliability of their input data for deep learning models.

## Introduction

Biologists use aerial observer surveys to estimate abundances of wildlife populations that occur in moderate to large congregations, inhabit remote areas, or are widely distributed (e.g., colonial waterbirds, ungulates, and marine mammals) [1–3]. Low-altitude aerial observer surveys can be resource intensive, costly, and risky [4], but these problems may be mitigated via imaging surveys using either unoccupied aerial systems (UAS) or occupied aircraft at higher altitude [5]. Additionally, aerial imaging shows promise in mitigating observer and detection biases by producing a digital record of the survey frame [5]. However, aerial imaging produces large volumes of data that can be time-prohibitive to process manually [6]. Artificial intelligence approaches in computer vision, particularly deep learning using convolutional neural networks (CNNs), have been successfully deployed to locate and count animals in aerial imagery [7]. CNNs must be trained to identify target species, which typically requires hundreds to thousands of annotated example images produced by human observers [8]. The substantial time commitment required to produce suitable pools of training data for deep learning is therefore an important obstacle for applying automated animal detection to aerial imaging for routine wildlife surveys.

Crowdsourcing has been successfully used to extract image information for training CNNs in computer vision tasks such as classification, object detection, and semantic segmentation [9]. For wildlife applications, researchers have also recruited the general public to volunteer to crowdsource image annotations via citizen science web platforms, in contrast to paid crowdsourcing image annotation platforms such as Mechanical Turk [10]. By distributing the annotation workload among large groups of volunteers, crowdsourcing can quickly produce large amounts of training data for CNNs. However, customized data filtering and/or aggregation protocols are often necessary to correct for the higher levels of observation error in crowdsourced data, and collection protocols must be carefully designed to ensure that data quality meets project objectives [11]. While volunteers appear to reliably detect [12] and enumerate animals to within approximately 10% of expert counts (with appropriate data quality control protocols) in aerial imagery [13], their ability to accurately classify different wildlife species from aerial imagery in a multispecies context has not yet been fully examined. The ability of volunteers to identify multiple terrestrial mammal species from camera trap images, including visually similar species and/or in complex environments, has been validated across variable geographic contexts [10, 14, 15]. However, classification of objects from aerial imagery is a unique and learned skill.

Aerial imagery presents challenges for image annotation even for experienced observers [16], particularly in complex environments [17]. It is unclear whether attributes of aerial images—such as spatial resolution, texture, and the number/distribution of targets of interest—may be related to interpretation difficulty. Kraff et al. [17] found that conflicting annotations of imagery of a complex urban environment were largely related to individual observer variability, with differences increasing with the complexity of the urban landscape; however, it is unclear whether image characteristics influence accurate distinction of wildlife, particularly in complex natural environments.

Concerns have been raised generally in the literature of deep learning about the validity of data used to train and test these models and the implications of imprecision and inaccuracy of inputs on the validity of model outputs [18–20]. Error in training and test data can yield misleading conclusions on model accuracy, even if initial validation metrics appear otherwise favorable [18, 19]. For example, biologists found substantial errors in the wildlife annotations incorporated into the benchmark ImageNet 1k dataset, including incorrect species identifications (12%), poorly defined and overlapping classes (11%), and a variety of unsuitable examples (e.g., plush toys, artistic depictions of animals) [18]. Without review, these errors may never become apparent when present across data splits for testing, training, and validation, particularly within the test set [19]. The wildlife data incorporated into ImageNet 1k were not assessed by biologists during the development of the dataset, and errors persisted despite review by multiple human observers during image annotation [18], reinforcing the requirement for properly vetting crowdsourced data prior to model development. To address these reliability concerns, a framework for establishing the accuracy and validity of image annotations is warranted prior to incorporating them into deep learning models—particularly for complex interpretation tasks such as identifications of wildlife from aerial images.

The average level of agreement among human observers for wildlife identification and counts can be used both to validate the performance of observers and to establish appropriate expectations for the accuracy achievable by a deep learning model trained using these data, given that models are typically bound by the precision of their inputs [21]. Because independently verified count and classification data are typically unavailable in wildlife imaging applications [3], agreement is a valuable proxy for assessing accuracy.

Our objective in this study was to provide a framework for assessing the reliability of image annotations prior to incorporation in a deep learning model by quantifying observer agreement. This framework has the additional benefit of providing a method for aggregating crowdsourced annotations of aerial imagery. To demonstrate this framework, we analyzed agreement among experts and volunteers annotating a small set of UAS images of a complex, multi-species environment that we considered a difficult scenario for both wildlife detection and classification due to the presence of multiple confounding factors such as shadow, occluding vegetation, mimicry, and camouflage. We investigated whether 1) agreement was present within each group (i.e., if experts agreed with each other, and volunteers agreed with each other); 2) agreement was present between the two groups (i.e., whether volunteers could produce data of similar quality to experts); and 3) image attributes contributed to observer agreement (or the lack thereof).

## Materials and methods

We examined agreement in counts and classifications of waterbirds by species or by morphological class (crane, duck, goose) in two sets of annotated unoccupied aerial system (UAS) imagery. Image data were collected at four wildlife management areas in New Mexico, USA. One set of image annotations was generated by professional wildlife biologists and the other by volunteers.

### Study area

**Fig 1:**
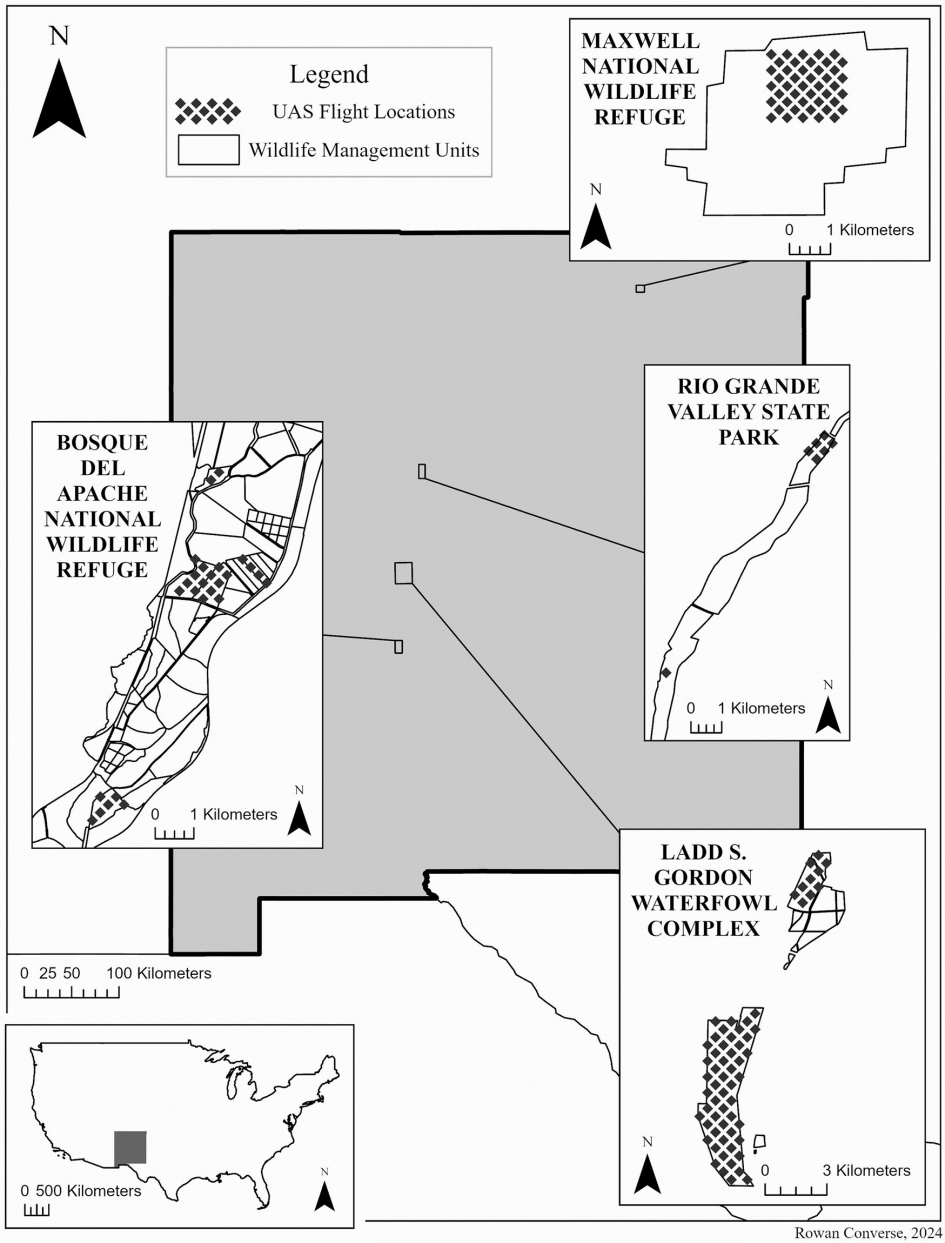
Map of the study area in New Mexico, USA. The waterfowl management units imaged via UAS are indicated with black diamond hatching.

The Middle Rio Grande Valley of New Mexico serves as a critical flyway for migratory birds and is host to a variety of government and private lands devoted to wildlife conservation. Our core study area was bounded by the city of Albuquerque to the north and Bosque del Apache National Wildlife Refuge to the south, with one ancillary site in northern New Mexico, Maxwell National Wildlife Refuge (Fig 1). The climate is semiarid, with an average annual precipitation of 240 mm. Average elevation is approximately 1,500 m above sea level. Riparian, lacustrine, wetland, and agricultural sites were sampled containing a mixture of open water, grass, forest, short-cycle crop, and woodland vegetation. Active management interventions to support migratory waterfowl and their habitat, such as cropping and controlled flooding, were employed at most sites by land managers.

### Data collection

#### Imagery

Natural color (RGB) UAS imagery of waterfowl was collected at state and federally managed wildlife areas in New Mexico from November to January between 2018–2022 (Fig 1). Twenty flights were conducted using a DJI Mavic Pro 2 sUAS equipped with a Hasselblad L1D-20c sensor (1”, 20MP, image size 5472 x 3648 pixels). Image resolutions ranged from 0.51 cm/px to 2.0 cm/px, with an average of 0.87 cm/px. All flights were conducted with permission from the appropriate land management agency and complied with agency policies, FAA Part 107 regulations, and best practices for imaging wildlife with drones [22]. See S1 Table for metadata on individual flights.

#### Expert image annotations

Fifteen biologists with waterbird survey experience from the US Fish and Wildlife Service, Texas Parks and Wildlife, and Ducks Unlimited annotated a twelve-image subset of UAS imagery collected at Bosque del Apache National Wildlife Refuge in 2018. Annotations were collected through the online image annotation platform Labelbox (https://www.labelbox.com). The twelve benchmark images were manually selected in consultation with biologists familiar with the study area (who were not recruited as annotators) to maximize species diversity and included a variety of backgrounds with varying vegetation and habitat types. At least one image was selected from each unique wildlife management unit included in the image surveys. A twelve-species classification scheme was derived from a previous survey at the site and refined via consultation with biologists familiar with the study area. Each observer drew polygons around individual birds and selected a species classification from the twelve options. Collectively, over 19,000 individual annotations of twelve waterfowl species were generated, with a redundancy factor of about 10x, given some incomplete annotations.

#### Crowdsourced image annotations

Crowdsourced annotations were collected through the participatory science platform Zooniverse (https://www.zooniverse.org; [23]). Established in 2007, Zooniverse is a web platform where researchers can upload visual or audio data for annotation by volunteers. The platform advertises new research projects to volunteers on the main page of the site and via an email list. As of publication, the site has over 2.7 million registered users. Zooniverse hosts projects across various disciplines including astronomy, ecology, art, architecture, social sciences, and medicine. The platform has been the source of several large and widely used annotated wildlife image datasets used for deep learning applications, including Snapshot Serengeti [10].

Users annotated UAS imagery of waterfowl using a simplified four-class morphology-based scheme by drawing rectangles around individual birds after viewing a brief tutorial with examples of the different classes. Though the expert group identified species, the classification scheme was collapsed to morphologically-based categories after feedback during initial testing indicated that the twelve-class scheme was untenable for volunteers who self-identified as having waterfowl species identification skills for reasons such as the unfamiliar vertical view angle, insufficient image resolution, and unfamiliarity with some of the species. For the first image upload in spring 2021 (consisting of imagery from 2018), the four-class scheme presented to users consisted of duck, goose, crane, and “other bird” to align with options presented to the expert group. In the second image upload in spring 2022, we removed “other bird” as a category to correspond with best practices for participatory science data to avoid overrepresentation of false negatives [24]. We added an additional category, gull, which was a taxon not present in the 2018 imagery but common at sites imaged in 2021 and 2022.

Each image was annotated by fifteen volunteers prior to being retired (i.e., considered to have received sufficient annotation), resulting in individual annotation redundancy of about 10x throughout the dataset due to differences in individual volunteers’ annotations. To increase detection likelihood, images of waterfowl were tiled prior to upload to increase the relative area of the image occupied by an individual bird. Each image was sliced into 56 tiles of 684 x 521 pixels [25] and after the first upload, only tiles containing birds were uploaded to reduce user fatigue. Images without birds were removed using a deep learning model trained on the expert annotations [25], followed by manual examination to remove any remaining empty images. We collected a total of 1,449,301 individual annotations from 4,351 Zooniverse volunteers of 30,951 image tiles derived from 1,032 UAS images.

### Data processing

**Fig 2:**
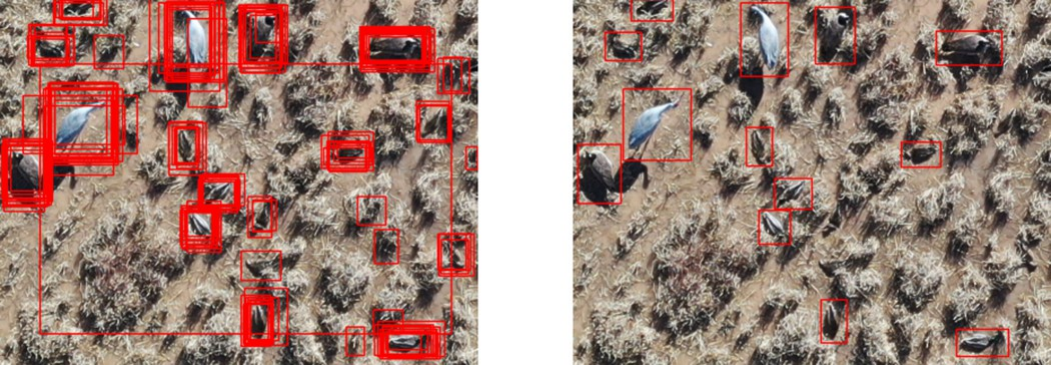
Annotation aggregation. Example image with raw, redundant annotations on the left, and aggregated annotations on the right. The right half of the figure is reprinted from [26] under a CC BY license with permission from the authors, original copyright 2023.

From the redundant image annotations generated by each group of observers (experts and volunteers), we derived a set of aggregated image annotations describing the location and species or morphological class of each individual bird within each image (Fig 2). The goal of the aggregation process, described below, was to capture the crowd’s agreement to provide a point of comparison for the performance of individual observers. Because there was no known number/identifications of birds to compare the observers’ performance against (as is typical in wildlife surveys), we instead compared 1) the performance of individual observers against their group’s aggregate, and 2) the aggregate annotations of each group against each other. Additionally, the aggregated annotation set represents the product that would be used to train a deep learning model, as redundant annotations of the same objects would be confusing for model learning.

To derive the aggregated annotation sets, we used the Density-Based Spatial Clustering of Applications with Noise (DBSCAN) algorithm [27] to associate groups of bounding boxes annotated by different observers with individual animals. The key parameter in this method is the maximum distance (ε) between neighboring bounding box center points for them to be considered part of the same cluster. We set this distance at 15 pixels, which we arrived at by iterating a k-distance nearest neighbor order calculated on a set of example annotations from ten random images in the crowdsourced dataset and the twelve images labeled by experts. We compared the counts produced via clustering with counts produced by the researchers, due to the lack of an independently known reference for the true number of animals in a given image. The minimum cluster size (i.e., minimum number of bounding box centers to define a cluster) was set at 5 after a similar iterative process. Bounding boxes that could not be associated with a cluster were excluded from the aggregated annotations. These excluded bounding boxes typically represented misidentifications such as confusing vegetation (e.g., bunch grass) as birds. Another minor source of error was bounding boxes apparently drawn over non-target objects such as open water and not deleted for unknown reasons. In rare cases, some unassociated bounding boxes appeared to be genuine bird detections that were only labeled by one or two observers.

We derived an aggregate bounding box for each cluster by calculating the median of each coordinate (top left x, top left y, width, height) of the bounding boxes within in each cluster. The morphological or species class for each aggregate bounding box was determined by taking the mode of all class annotations within in the cluster. The mode was used in place of the majority to allow for a classification decision on “difficult” targets where a majority classification may not occur among observers, validated in other volunteer crowdsourcing species classification contexts [24]. For each individual bounding box in a cluster, we calculated an intersection over union (IOU) with the corresponding aggregate bounding box. This metric quantifies agreement between bounding box dimensions and locations; an IOU threshold of 0.5 is commonly used to confirm object detection success [28]. We used this metric as an indication of likelihood that each of the individual bounding boxes within a cluster corresponded to detections of the same animal.

### Analysis

#### Assessment of observer agreement

We assessed observer agreement both within each group (i.e., among experts, among volunteers) and between the two groups (experts vs volunteers). To assess agreement within groups, we compared each individual observer’s annotations against the set of aggregated annotations for that group. Any discarded annotations (i.e., those not associated with a cluster) were compared to a null value to ensure automatic failure with the comparison criteria. Identifications in the expert labels were evaluated at two categorical scales: at the species level, and as aggregated groups based on morphology (i.e., duck/goose/crane), while identifications in the crowdsourced labels were evaluated at only the morphological scale.

We calculated average agreement with the aggregated annotations both per user and across users for each class. Additionally, following the method proposed by Swanson et al. [24] for assessing agreement among redundant volunteer classifications of wildlife, an adaptation of Pielou’s evenness index [29] was calculated for each aggregate label. The index was calculated as:

$$-(\sum_{i=1}^{S}p_{i}\;\text{ln}\;p_{i})/\text{ln}\;S$$

where *S* is the number of class identifications by observers associated with an individual animal, and *p*_*i*_ is the proportion of identifications of a given class *i*. Values closer to 1 are interpreted as representing higher disagreement (i.e., more classes identified by observers), and values closer to 0 representing greater agreement (i.e., fewer classes identified by observers). When only one class was identified by all observers, we re-scored the index as zero to allow for a wider distribution of values that would be easier to interpret [24].

To assess agreement between the expert and volunteer groups, we compared the aggregated crowdsourced annotations to the aggregated expert annotations for the twelve-image benchmark set labeled by both groups. For this analysis, the expert annotations were split into tiles corresponding to the tiles labeled by the volunteer group to ensure that counts generated by each group were comparable. Count agreement overall and by class were compared. To assess agreement on localizations of individual birds within the images, a confusion matrix was calculated using the expert labels as the reference and the crowdsourced labels as the predictions at an intersection over union (IOU) threshold of 0.5 [28].

#### Image attributes and observer agreement

To assess whether measurable image attributes were associated with observer agreement in both groups, a binomial logit model relating image attributes to agreement was constructed separately for the experts and the volunteers. For this analysis, the proportion of identifications for an individual annotation that agreed with the aggregate class identification was regressed against a set of image attribute covariates (Table 1). To mitigate issues with multicollinearity, we iteratively calculated a variance inflation factor (VIF) in both aggregate annotation sets for the image factor covariates, removing those with a score >5 until the remaining covariates scored below the threshold.

**Table 1:** Descriptions of image attribute covariates that were assessed for inclusion in the binomial logit models relating these characteristics to observer agreement.

Variable	Description	Included in Expert Binomial Logit Model?	Included in Crowdsourced Binomial Logit Model?
**Area**	Area of aggregate bounding box in pixels	No	No
**Bounding box percent area**	Area of the aggregate bounding box as a percent of total image area	Range: 0.002–0.168 Mean: 0.016; SD 0.016	Range: 0.009–17.041; Mean: 1.142; SD: 0.732
**Same class percent**	Percent of other aggregate annotations in the image assigned the same class identification	No	No
**Number of neighbors**	Number of aggregate bounding boxes within a radius defined as 2x of the greater of the width or height of the consensus bounding box	Range: 0–11; Mean: 1.77; SD: 1.557	Range: 0–35; Mean: 1.364; SD: 1.41
**AGL**	Image capture altitude above ground level of the UAS, in meters	No	No
**Spatial Resolution**	Spatial resolution of the image in centimeters per pixel	No	No
**Distance from center**	Distance between the center of the aggregate bounding box and the image center, in pixels	Range: 0.389–30.255; Mean: 13.05; SD: 6.698	No
**Density**	Total number of other aggregate annotations in the image	Range: 23–722; Mean: 411.004; SD: 253.415	Range: 1–64; Mean: 11.213; SD: 10.322
**Rarity**	Percent of other annotations in the total annotation dataset that belong to the same class. Only calculated for the expert annotations.	No	No
**Bounding box GLCM covariates**	Image texture measure [30]: Gray-Level Co-occurrence Matrix values (contrast, energy, dissimilarity, homogeneity) calculated for pixels within a aggregate bounding box using 1, 3, and 5-pixel offsets across eight angles.	No	No
**“Donut” GLCM covariates**	Gray-Level Co-occurrence Matrix values calculated as above, for 20-pixel interior + exterior buffer from an individual aggregate bounding box to capture a transition area between the target and its immediate surroundings due to the irregular shape of a target compared to a rectangular bounding box	No	No
**Bounding box / donut GLCM difference covariates**	Simple difference between bounding box and “donut” GLCM covariates (contrast, energy, dissimilarity, homogeneity)	Only energy difference included Range: -0.019–0.071; Mean: 0.0003; SD: 0.01	Only energy difference included Range: -0.149–0.193; Mean: 0.002; SD: 0.009
**Species/morphological class covariates**	Dummy variables for species class (expert) or morphological class (crowdsourced)	Yes, but excluded Other	Yes, but excluded Gull and Other

Image and annotation covariates assessed for inclusion in a logistic regression model for examining impacts of image attributes on observer agreement. Covariates excluded due to high multicollinearity as measured by VIF scores were not included in the model. Basic statistics (range, mean, SD) of included variables are given.

See S1 File for a link to our analytic code.

## Results

### Expert agreement

Of the 19,336 annotations generated by experts, 1,238 (6%) were not in close enough proximity to at least five other annotations, the threshold for association with a cluster, and were discarded. An aggregated set of 2,375 annotations was produced through the clustering process (Table 2). From this total, 132 aggregate bounding boxes (5.5%) were discarded for species identification because a plurality vote on species class was not reached: i.e., there were equal numbers of votes for two or more species classes. These annotations were preserved at the morphological class level, as this type of error only resulted between duck species.

**Table 2:** Expert annotation counts by species and morphological class, pre- and post- aggregation.

Morphological Class	Aggregate Annotation Count	Raw Annotation Count	Species Class	Aggregate Annotation Count	Raw Annotation Count
**Duck**	2,111	15,915	Mallard (*Anas platyrynchos*)	1,688	11,775
			Northern Pintail (*Anas actua*)	262	2,369
			American Wigeon (*Mareca americana*)	22	621
			Teal (*Anas discors*)	2	518
			Gadwall (*Mareca strepera*)	5	368
			Northern Shoveler (*Spatula clypeata*)	2	183
			Ringneck (*Aythya collaris*)	0	35
			Redhead (*Aythya americana*)	0	25
			Ruddy Duck (*Oxyura jamaicensis*)	0	21
			Equal votes for two or more species	132	n/a
**Goose**	140	1,143	Canada Goose (*Branta canadensis*)	140	1,140
			Snow Goose (*Chen caerulescens*)	0	3
**Crane**			Sandhill Crane (*Grus canadensis*)	52	516
**Other**	70	1,762			

Aggregate expert annotations by species and morphological class compared to raw annotation counts (i.e., all individual observer annotations added together).

The average observer agreement with the aggregated species-level identifications was 0.74 (SD 0.43), with substantial variation between individual observers (Table 3). The average Pielou index across annotations was 0.29 (SD 0.35), with large differences between average scores among the species classes (Table 3). Duck species classes tended to have high Pielou index scores (i.e., low agreement), particularly among the species with relatively few aggregate annotations in the dataset. Agreement was high for Canada Goose and Sandhill Crane identifications, with average Pielou indices of 0.24 (SD 0.26) and 0.01 (SD 0.07), respectively. The average IOU between individual bounding boxes and the corresponding aggregate box was 0.63 overall. IOU averaged between 0.61 and 0.69 across the different species.

**Table 3:** Individual expert agreement with the aggregated expert annotation set.

Observer ID	Overall	Sandhill Crane	Canada Goose	Mallard	Northern Pintail	Northern Shoveler	American Wigeon	Gadwall	Teal	Other	Total Annotations
**# Aggregated Annotations**		52	140	1688	262	2	22	5	2	70	2,243
**1**	0.83	1.00	1.00	0.99	0.88	1.00	0.50	0.60	0.50	0.02	1,145
**2**	0.72	1.00	1.00	0.87	0.72	0.00	0.18	0.00	1.00	0.88	2,315
**3**	0.75	1.00	1.00	0.88	0.83	0.50	1.00	0.50	0.00	0.25	1,983
**4**	0.77	1.00	1.00	0.77	0.93	0.00	0.57	0.25	1.00	0.97	1,395
**5**	0.68	0.98	0.98	0.85	0.82	0.50	0.83	0.80	1.00	0.08	2,747
**6**	0.82	1.00	1.00	0.98	0.89	1.00	0.21	0.50		0.13	1,791
**7**	0.45	1.00	1.00	0.44	0.09	0.00	0.50	0.00	0.00	0.84	1,076
**8**	0.85	1.00	0.91	0.96	0.92	0.00	0.50	0.00		1.00	1,426
**9**	0.98	1.00	0.75	0.99	0.99	0.00	0.00	0.00		0.00	508
**10**	0.42	1.00	0.61	0.40	0.67	1.00	0.78	0.80	0.50	0.02	1,844
**11**	0.83	1.00	0.99	0.92	0.35	1.00	0.29	0.00	0.50	1.00	960
**12**	0.96	1.00	1.00	0.99	1.00		1.00	0.00		0.00	485
**13**	0.92	1.00	1.00	0.94	0.80		1.00	0.00		0.50	485
**14**	0.95	1.00	1.00	0.98	0.86		0.50	0.00		0.60	1,096
**15**	0.93	1.00	1.00	0.73	1.00						68
**Average Agreement**	0.74 (SD 0.43)	0.99 (SD 0.05)	0.93 (SD 0.24)	0.83 (SD 0.37)	0.81 (SD 0.38)	0.46 (SD 0.51)	0.56 (SD 0.50)	0.43 (SD 0.50)	0.53 (SD 0.51)	0.47 (SD 0.50)	19,336
**Average Pielou Index**	0.46	0.01	0.25	0.43	0.48	0.92	0.83	0.91	0.89	0.90	
**Average IOU**	0.63	0.68	0.61	0.63	0.69	0.64	0.67	0.67	0.69	0.62	

Individual observer agreement with aggregated annotations by species, with average Pielou index and average IOU* of individual bounding box vs aggregate box. Pielou indices closer to 0 indicate higher levels of agreement.

*IOU = Intersection over Union

The average individual agreement with the aggregate classification for morphological-level classifications was notably higher than the species-level classifications. For the three classes of interest, average agreement with the aggregate classification was 0.93 (SD 0.26) for Duck, 0.94 (SD 0.24) for Goose, and 0.99 (SD 0.05) for Crane (Table 4). The average Pielou index for the aggregated Duck class (0.25, SD 0.32) was much lower (i.e., higher agreement) than for any given duck species class from the species-level analysis.

**Table 4:** Expert annotation agreement metrics by morphological class.

Morphological Class	Average Agreement	Average Pielou Index	Average IOU*
**Duck**	0.93 (SD 0.25)	0.25 (SD 0.32)	0.66
**Goose**	0.94 (SD 0.25)	0.24 (SD 0.26)	0.61
**Crane**	0.99 (SD 0.04)	0.01 (SD 0.06)	0.68
**Other**	0.47 (SD 0.49)	0.96 (SD 0.11)	0.62
**All Classes**	0.83 (SD 0.43)	0.30 (SD 0.35)	0.63

Metrics comparing the average of individual expert annotations with the consensus expert annotations by morphological class: includes average agreement, average Pielou index, and average IOU.

*IOU = Intersection over Union

### Volunteer agreement

Of the 1,449,301 crowdsourced annotations, 196,693 (13.5%) could not be associated with a cluster and were discarded. The clustering process produced an aggregate set of 150,307 annotations (Table 5). Of this set, 2,950 aggregate bounding boxes (2%) were discarded because a plurality vote on class identification was not reached, yielding a usable total of 147,357 aggregate annotations. The most frequent tie vote leading to lack of agreement in the morphological categories occurred between duck and goose (n = 1,243), followed by crane and duck (n = 284), and crane and goose (n = 213). There were also tie votes between duck and gull (n = 758), and infrequently, gull and crane (n = 14) or goose (n = 36). The remaining sources of tied votes were between any of the categories and “Other”, or equal votes for all categories.

**Table 5:** Crowdsourced annotation counts by morphological class, pre- and post-aggregation.

Morphological Class	Raw Annotation Count	Aggregate Annotation Count	Average Agreement	Average Pielou Index	Average IOU*
**Duck**	873,553	92,286	0.92 (SD 0.25)	0.22 (SD 0.31)	0.68
**Goose**	298,253	31,760	0.74 (SD 0.44)	0.71 (SD 0.25)	0.69
**Crane**	115,685	8,886	0.95 (SD 0.21)	0.18 (SD 0.27)	0.72
**Gull**	152,538	14,350	0.91 (SD 0.27)	0.28 (SD 0.32)	0.72
**Other**	9,272	75	0.52 (SD 0.50)	0.86 (SD 0.16)	0.69
**No ID (tie vote)**	n/a	2,950	n/a	n/a	n/a
**TOTAL**	1,449,301	150,307	0.74 (SD 0.43)	0.27 (SD 0.35)	

Crowdsourced annotation counts by morphological class, along with average observer agreement with the aggregate classification, average Pielou index and average IOU* between individual annotators’ bounding boxes and the corresponding aggregate box.

*IOU = Intersection over Union

The average agreement among individual crowdsourced observers compared to the crowdsourced aggregated annotations was 0.75 (SD 0.43). For the three classes of interest, average agreement with the aggregated classification was 0.95 (SD 0.21) for Crane, 0.74 (SD 0.44) for Goose, and 0.92 (SD 0.25) for Duck (Table 5). The average Pielou index across all annotations was 0.27 (SD 0.25), and for the three classes of interest was 0.18 (SD 0.27) for Crane, 0.22 (SD 0.31) for Duck, and 0.71 (SD 0.25) for Goose (Table 5). Average IOU between individual bounding boxes and the aggregate box was 0.69, ranging from 0.68 to 0.72 depending on morphological class.

### Expert vs. Volunteer agreement

**Fig 3:**
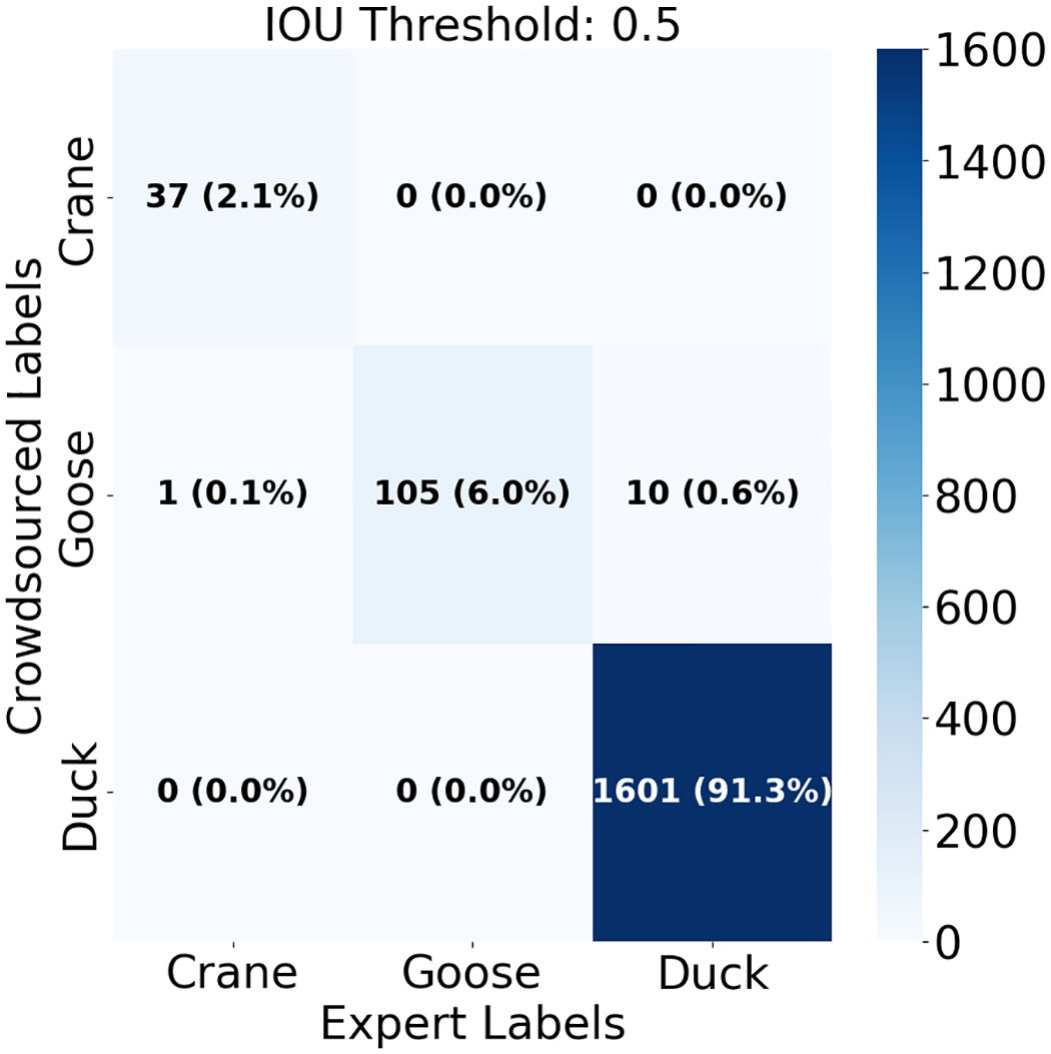
Expert/crowd annotation agreement. Confusion matrix describing the agreement between the image location of the expert and crowdsourced annotations using an IOU threshold of 0.5. The integer value is the number of matching annotations of a given class, followed by the percentage that class represented in the matching annotations.

We compared the aggregated annotations from the crowdsourced set and the expert set, considering the expert annotations as the reference set. When pooling all birds together into a generic class (“bird”), across all the images, the total number of birds identified in the aggregated crowdsourced annotations was 91% of the total number of birds identified in the aggregated expert annotations. The total count for each morphological class from the aggregated crowdsourced annotations was 89% of the expert count for crane, 80% for goose, and 91% for duck. The count of birds across individual image tiles was not significantly different between the two groups (t = 1.27, df = 338, p = 0.20). The range of count differences was highest for the duck class (-9 to +25), while the range for geese was -4 to +2 and the range for crane -1 to +2. Locations of individual bird annotations within the images matched 81%, and when locations matched, identifications of morphological classes matched 99.4% (Fig 3).

### Image attributes analysis

We report significant odds ratios from binomial logit models relating image attribute covariates to observer agreement for experts and volunteers respectively.

Among experts, bounding box size relative to image area, differences in image texture between target and background, and number of neighboring targets had the greatest positive influence on agreement, while distance of the target from the image center had a negative impact on agreement. For each 1% increase in the percent area of the bounding box relative to the image size, the odds of agreement increased 1.662 times (*e*^50.825/100^, *β* = 50.825, SE = 3.594, *P* < 0.001). The odds of agreement increased 9.8% for each 0.01 increase in texture (as represented by a Gray-Level Covariance Matrix energy calculation) difference between a target and its immediate background (*e*^9.357/100^, *β* = 9.357, SE = 2.395, *P* < 0.001). The odds of agreement increased 15.5% for each incremental increase in the number of neighboring annotations (*β* = 0.144, SE = 0.017, *P* < 0.001). There was a 1.5% decrease in odds of agreement with each pixel increment of distance of the annotation from the center of the image (*β* = –0.015, SE = 0.004, *P* < 0.001).

In contrast, among volunteers, differences in image texture between the target and the background had a negative influence on agreement, the total number of targets in the image had a positive effect, and other variables had negligible effects. For every 0.01 increase in textural difference between target and background, the odds of agreement decreased 12.4% (*e*^−13.222/100^ *β* = -13.222, SE = 0.371, *P* < 0.001). With each additional annotation in the image, there was a 2% increase in odds of agreement (*β* = 0.02, SE = 0.001, *P* < 0.001).

## Discussion

Our methods provide a reproducible framework useful for practitioners implementing aerial imaging surveys for wildlife monitoring. The data aggregation method we described provides both 1) a single annotation set that can be used as an analytic product for counts/identifications of wildlife and for training deep learning models and 2) a point of comparison against which individual observer performance can be assessed in the absence of an independent reference. We found that agreement metrics provide a reliable aggregate assessment of the performance within a group of observers as well as a suitable comparison between volunteer and expert observers. While we had 15x observer redundancy in our study (following recommendations from the citizen science literature [24]), this level of redundancy may not always be realistic particularly for experts, who can be hard to delegate or have limited availability. However, as we explain below, it is likely that agreement metrics can be derived with fewer redundant annotators.

For our test case, our results show that, in aggregate, experts and volunteers were capable of producing similar quality annotations of high-resolution aerial imagery of waterfowl. This is in spite of our study area representing a relatively difficult interpretation scenario due to the presence of multiple confounding factors such as shadow, occluding vegetation, and camouflage. The aggregated annotation sets from each group agreed with each other closely (within 9% for overall counts and >99% for morphological class identifications). The level of classification agreement among members of the same group was broadly similar for both experts and volunteers, although volunteers had notably lower agreement (75%) amongst themselves for classifications of geese than experts. Because of this finding, we recommend that imagery presented to observers uses a constant scale to avoid confusion between size classes. In addition, using a minimum aggregation of five observer annotations was effective for crowdsourcing multi-class aerial image annotations to mitigate class-specific variability, five being the minimum number of annotations we found necessary to cluster bounding boxes during the data aggregation process. However, the strong consistency between experts for morphological classifications across classes indicates that redundancy is not necessary when sourcing annotations from experts at this taxonomic level. Therefore, our assessment confirms that roughly five volunteers can in aggregate produce data of similar quality to one expert.

We found that neither observer group could adequately identify duck species (geese and cranes were represented by one species each in our imagery set). Volunteers self-selected out of the task during beta testing. While agreement among experts was relatively high for Mallard (83%) and Northern Pintail (81%), the very low agreement for some of species that were less numerous in the imagery, but not generally uncommon, such as Gadwall (43%), Northern Shoveler (46%), and American Wigeon (56%), casts doubt on the validity on the general set of duck species identifications. Additionally, three duck species (Ringneck, Ruddy Duck, and Redhead) were completely eliminated from the aggregated annotation set due to none of the individual observers’ annotations for these species achieving a plurality vote for any single bird annotation. While our image sample size was limited, we believe our findings provide an important outcome for other practitioners to consider due to the relatively high number of experts we were able to recruit and the fact that most of the species that had high disagreement are relatively common (i.e., we would expect experts to be familiar with them). However, the nadir angle may obscure features that would make visually similar species distinguishable from other angles. For instance, Northern Shoveler, a species with low agreement in our study, may appear similar to Mallard from above due to similarities in coloration and size, but more distinguishable from the side due to differences in bill shape that may be difficult to distinguish from above. It is possible this points to fundamental limitations of the information that is provided at the spatial resolution of the imagery from our sensor, which is similar to the resolution of imagery from imaging arrays used in aerial imaging survey programs in the United States [31]. It is possible that increased spatial resolution could help experts resolve difficulties in duck species identification; for instance, Dulava et al. [32] found that a minimum spatial resolution of 5mm per pixel was required for positive differentiation of known decoys of duck species at their sites in similar habitats with many of the same species as our study area (e.g., Mallard, Northern Pintail, Northern Shoveler, American Wigeon, Gadwall). However, achieving this resolution while also maintaining sufficient flight altitude above waterfowl to avoid disturbance [22] is difficult with most sensors currently available to the consumer market. It is worth noting that species-level classifications may not be necessary for all management objectives [33]. Therefore, aerial methods may remain useful for waterfowl monitoring despite limited ability for species-level classifications of ducks.

While the explanatory power was limited for the logit models of image attribute covariates’ impact on observer agreement, these models provided insights that are relevant to developing an image annotation strategy for training deep learning models. Overall, tiling images prior to presenting them to users for annotation seemed to help increase detection and class agreement. For experts, the size of the bounding box relative to the total image size was by far the strongest factor associated with greater classification agreement. Experts labeled full-size images, where each individual target represented <1% of total image area, making any increase in target size have a much greater impact on detectability. In contrast, volunteers labeled image tiles, where the relative target size to image size was greater, making detectability based on size less important. Additionally, among experts, the number of immediate neighboring aggregated annotations positively influenced the likelihood of agreement, while the overall number of aggregated annotations in an image (regardless of location) did not; however, again, these effects were negligible for volunteers. For experts, clusters of birds likely increased detectability in the full-size images, where individual targets covered a relatively smaller area. Given the smaller size of the image tiles and the relative larger size of individual birds compared to image size, there was less of a contrast for volunteers between clusters of birds, and images that were dense with birds overall.

## Conclusion

This study provides a framework for determining the reliability of human observer annotations from aerial image wildlife surveys. We present a method for aggregating redundant observer annotations which can be used to train deep learning models and also as a means to assess agreement among observers with different levels of experience. We used agreement as a proxy for accuracy due to the lack of an independent reference for wildlife counts and identifications, as is common in wildlife studies. To demonstrate our framework, we analyzed agreement between volunteers and experts annotating UAS imagery of waterfowl in complex, multi-species environments in New Mexico that represent a difficult case both for image interpretation and for deep learning. We found that the two groups in aggregate produced annotations of similar quality; counts agreed 91%, locations of animals agreed 81%, and when locations matched, classifications matched 99.4%. Both groups generally had high (92–99%) levels of agreement among themselves for classifications of broad morphological classes of birds (duck/goose/crane), except among volunteers labeling geese (75%). We found that aggregating multiple observer annotations is likely a necessary pre-processing step when using crowdsourced annotations before developing and evaluating automated wildlife detection models. We were not able to obtain reliable annotations of duck species: experts could not agree and volunteers opted out of the task. These results suggest that there is a limit on the taxonomic resolution achievable via aerial methods using current technology, but further study is warranted due to our limited sample size. Our analysis of the impacts of image attributes on wildlife classification agreement suggests that utilizing a constant scale and subsetting images to increase the size of targets relative to total image area during annotation may help increase agreement among individual observers and reduce classification errors common to each observer group. These findings can inform efforts to develop suitable inputs to deep learning models with the goal of automating aerial image processing in wildlife surveys, while tempering expectations regarding the reliability and range of applications suitable for suitable for these techniques.

## Supporting information

S1 Table UAS survey metadata. (DOCX)

S1 File Link to analysis code. (DOCX)
