# Supplementary material for: A framework for assessing reliability of observer annotations of aerial wildlife imagery, with insights for deep learning applications
Source: PLoS One. 2025 Jan 15;20(1):e0316832. doi: 10.1371/journal.pone.0316832 (PMC11734989; doi:10.1371/journal.pone.0316832)
Supplement: S1 Table — (DOCX) [file pone.0316832.s001.docx]

| **Location** | **Date** | **Start Time** | **# Images** | **Flight Altitude (m AGL^1^)** | **GSD^2^ (cm/px)** |
| --- | --- | --- | --- | --- | --- |
| Maxwell NWR- Lake 13 | 12/15/2017 | 12:00 | 1963 | 60 | 1.38 |
| Bosque del Apache NWR- Unit 18/a03 | 11/6/2018 | 12:00 | 263 | 40 | 1.12 |
| Bosque del Apache NWR- Unit 18/a04 | 11/6/2018 | 13:00 | 304 | 30 | 0.76 |
| Bosque del Apache NWR- Unit 18/a04 | 11/6/2018 | 14:00 | 375 | 20 | 0.51 |
| Bosque del Apache NWR- Unit 18/b_w | 11/7/2018 | 9:30 | 160 | 20 | 0.51 |
| Bosque del Apache NWR- Unit 18/d | 11/7/2018 | 10:00 | 385 | 40 | 1.12 |
| Bosque del Apache NWR- Unit 18/d | 11/7/2018 | 11:00 | 157 | 30 | 0.76 |
| Bosque del Apache NWR- Unit 34/c | 11/7/2018 | 12:40 | 613 | 44 | 1.53 |
| Bosque del Apache NWR- Unit 12c/01 | 11/27/2018 | 11:30 | 518 | 40 | 1.12 |
| Atrisco | 12/05/2021 | 8:40 | 295 | 30 | 0.76 |
| Rio Grande State Park- Alameda Bridge | 12/12/2021 | 11:40 | 705 | 80 | 2.03 |
| Ladd S Gordon Wildlife Management Area: Bernardo | 01/10/2022 | 10:40 | 86 | 40 | 1.12 |
| Ladd S Gordon Wildlife Management Area: Bernardo | 01/10/2022 | 10:55 | 146 | 40 | 1.12 |
| Ladd S Gordon Wildlife Management Area: Bernardo | 01/10/2022 | 11:15 | 197 | 40 | 1.12 |
| Ladd S Gordon Wildlife Management Area: Bernardo | 01/10/2022 | 11:40 | 129 | 40 | 1.12 |
| Ladd S Gordon Wildlife Management Area: Bernardo | 01/11/2022 | 10:00 | 134 | 40 | 1.12 |
| Ladd S Gordon Wildlife Management Area: Bernardo | 01/11/2022 | 10:30 | 277 | 40 | 1.12 |
| Ladd S Gordon Wildlife Management Area: La Joya | 01/11/2022 | 12:30 | 220 | 40 | 1.12 |
| Ladd S Gordon Wildlife Management Area: La Joya | 01/11/2022 | 12:40 | 227 | 30 | 0.76 |
| Rio Grande State Park- I-40 Bridge | 01/29/2022 | 11:40 | 529 | 40 | 1.12 |

^1^AGL = Above Ground Level

^2^GSD = Ground Sampling Distance
