# Supplementary material for: A framework for assessing reliability of observer annotations of aerial wildlife imagery, with insights for deep learning applications
Source: PLoS One. 2025 Jan 15;20(1):e0316832. doi: 10.1371/journal.pone.0316832 (PMC11734989; doi:10.1371/journal.pone.0316832)
Supplement: S1 File — (DOCX) [file pone.0316832.s002.docx]

**S2. Link to Analytic Code:** <https://github.com/rowanoaktree/publications/tree/main/label_consensus>
